# Brain mechanisms of short-term habituation and sensitization toward dyspnea

**DOI:** 10.3389/fpsyg.2015.00748

**Published:** 2015-06-02

**Authors:** M. Cornelia Stoeckel, Roland W. Esser, Matthias Gamer, Christian Büchel, Andreas von Leupoldt

**Affiliations:** ^1^Department of Systems Neuroscience, University Medical Center Hamburg-EppendorfHamburg, Germany; ^2^Research Group Health Psychology, Faculty of Psychology and Educational Sciences, University of LeuvenLeuven, Belgium

**Keywords:** dyspnea, breathlessness, habituation, sensitization, fMRI, unpleasantness, insular cortex

## Abstract

Dyspnea is a prevalent and threatening cardinal symptom in many diseases including asthma. Whether patients suffering from dyspnea show habituation or sensitization toward repeated experiences of dyspnea is relevant for both quality of life and treatment success. Understanding the mechanisms, including the underlying brain activation patterns, that determine the dynamics of dyspnea perception seems crucial for the improvement of treatment and rehabilitation. Toward this aim, we investigated the interplay between short-term changes of dyspnea perception and changes of related brain activation. Healthy individuals underwent repeated blocks of resistive load induced dyspnea with parallel acquisition of functional magnetic resonance imaging data. Late vs. early ratings on dyspnea intensity and unpleasantness were correlated with late vs. early brain activation for both, dyspnea anticipation and dyspnea perception. Individual trait and state anxiety were determined using questionnaire data. Our results indicate an involvement of the orbitofrontal cortex (OFC), midbrain/periaqueductal gray (PAG) and anterior insular cortex in habituation/sensitization toward dyspnea. Changes in the anterior insular cortex were particularly linked to changes in dyspnea unpleasantness. Changes of both dyspnea intensity and unpleasantness were positively correlated with state and trait anxiety. Our findings are in line with the suggested relationship between the anterior insular cortex and dyspnea unpleasantness. They further support the notion that habituation/sensitization toward dyspnea is influenced by anxiety. Our study extends the known role of the midbrain/PAG in anti-nociception to an additional involvement in habituation/sensitization toward dyspnea and suggests an interplay with the OFC.

## Introduction

The experience of dyspnea (breathlessness) is the cardinal symptom in prevalent diseases such as asthma and chronic obstructive pulmonary disease (COPD). It is also common in other pathologies including cardiovascular and neuromuscular diseases, and panic and anxiety disorders (Parshall et al., 2012; Laviolette et al., 2014). Notably, the perception of dyspnea is not linearly related to objective lung function or sensory input. Instead, dyspnea perception has been shown to be modulated by several psychological factors, including attention, expectation, learning, categorization and comparison processes, emotional predispositions, and current mood (e.g., Janssens et al., 2009; Lansing et al., 2009; Herigstad et al., 2011; Petersen et al., 2011, 2014). Via their influence on dyspnea perception these factors also have a strong impact on coping strategies, disease management and disease progression (Hayen et al., 2013).

Furthermore repeated exposure to dyspnea can result in either increasing (sensitization) or decreasing (habituation) dyspnea perception (Bloch-Salisbury et al., 1996; Carrieri-Kohlman et al., 2001; Wan et al., 2008, 2009; von Leupoldt et al., 2011a; Hayen et al., 2015). Habituation toward dyspnea seems favorable in conditions such as COPD and panic disorder. In both conditions the experience of dyspnea might result in unfavorable avoidance behavior and habituation toward dyspnea might reduce this behavior. In COPD, in particular, avoidance of physical activity is one potential mechanism that accelerates disease progression as reduced physical fitness leads to unfavorable systemic consequences and has a negative effect on dyspnea severity ending in a spiral of decline (e.g., Troosters et al., 2013). In asthma patients, however, habituation to dyspnea might result in delayed treatment and critical under-medication (von Leupoldt et al., 2009, 2011a,b). Here, sensitization toward dyspnea seems more favorable as far as it might improve self-management by supporting the early initiation of actions during the onset of asthma exacerbations and by heightening the compliance with prescriptions.

Both habituation and sensitization toward dyspnea have been shown to interact with psychological factors such as negative affect. When healthy individuals repeatedly underwent hypercapnic rebreathing, more anxious individuals showed less habituation (Li et al., 2006). This reduction in habituation was more pronounced for the affective dimension of dyspnea (=unpleasantness) as compared to the sensory dimension of dyspnea (=intensity) (Wan et al., 2012).

Understanding the interplay of affective traits and -states with habituation vs. sensitization toward dyspnea seems crucial for the improvement of disease management and rehabilitation in those suffering from dyspnea. In this regard, a better knowledge of the underlying neural mechanisms appears important. However, the neural mechanisms involved in habituation vs. sensitization toward dyspnea have rarely been investigated (von Leupoldt et al., 2011a). In a previous study we observed reduced dyspnea unpleasantness ratings in patients with asthma to be related to reduced activations in the insular cortex and increased activations as well as gray matter volume in the anti-nociceptive periaqueductal gray (PAG) when compared with healthy controls (von Leupoldt et al., 2009, 2011b). These neural patterns were partly correlated with disease duration. Although these findings were interpreted as habituation toward dyspnea over time, the study did not directly examine habituation to repeated experiences of dyspnea.

In the present study, we repeatedly induced dyspnea in a set of healthy volunteers with parallel acquisition of functional magnetic resonance imaging (fMRI) data. We hypothesized that habituation/sensitization toward dyspnea unpleasantness as compared to dyspnea intensity would be correlated with activation in brain structures thought to be involved in the processing of dyspnea unpleasantness, in particular the insular cortex and the amygdala (von Leupoldt et al., 2008, 2009; Paulus et al., 2012). The amygdala has also been demonstrated to be involved in sensitization toward repetitive pain exposure (Stankewitz et al., 2013). Additional candidate areas were derived from the anti-nociceptive network involving the anterior cingulate cortex (ACC), and the midbrain/PAG (e.g., Petrovic et al., 2002; Bingel et al., 2007). A study investigating habituation toward aversive visceral stimulation demonstrated changes in the connectivity between rACC, and amygdala over repeated painful stimulation (Labus et al., 2009). A more recent study using repeated presentations of similar stimuli showed either increases or decreases of brain activation in the insular and cingulate cortex and the amygdala (Lowén et al., 2015).

We furthermore expected to see habituation/sensitization-related brain activation already during the anticipation of dyspnea. Although the anticipation of dyspnea has rarely been studied, one study on the anticipation of hyperventilation (Holtz et al., 2012) suggests an involvement of the orbitofrontal cortex (OFC) and the dorsomedial prefrontal cortex (dmPFC) along with insula and ACC. These areas also showed expectancy effects in placebo-studies on the anticipation/perception of pain (e.g., Hsieh et al., 1999) and were, therefore, of particular interest. Finally, we expected an interplay between anxiety-related personality traits and states with the dynamics of dyspnea ratings over late vs. early trials.

## Materials and Methods

### Participants

We re-analyzed data from 46 healthy individuals from a previous study (to be published elsewhere) with a specific focus on habituation/sensitization toward dyspnea. Normal lung function of participants (mean age 28.5 years, 18 females) was confirmed by standard spirometry (Miller et al., 2005). All participants negated any history of neurological, psychiatric, or respiratory disease. None of the subjects showed any anatomical anomaly of the throat. The average body-mass index was 23.4 (range 19.4–28.7). Trait- and state-anxiety were assessed using the State-Trait-Anxiety Inventory (STAI-T, STAI-S), Version X (Spielberger et al., 1983). Questionnaire data were analyzed after completion of the study. Written informed consent was obtained prior to the study. The study protocol was approved by the local medical ethics committee.

### Induction of Dyspnea and Measurement of Respiratory Parameters

Volunteers breathed through a tightly fitted face mask that was connected to a breathing circuit. Dyspnea was induced by the introduction of MRI-compatible resistive loads to the inspiratory end of the breathing circuit. In a pre-test before entering the scanner, subjects were placed in a supine position and presented with loads of increasing magnitude. We explained dyspnea to our participants as a sensation of difficult and uncomfortable breathing. Then, each load was presented for 24 s and dyspnea intensity subsequently rated on a Borg-scale (0 = “not noticeable” to 10 = “maximally imaginable”). Load magnitude was increased until subjects reliably reported a sensation of “severe” dyspnea (Borg Score > 5). The respective load was then used to induce severe dyspnea during scanning (mean/SD = 2.23/1.18 kPa/l/s). For the baseline condition of mild dyspnea the smallest resistive load that was reliably rated higher than unloaded breathing was used (mean/SD = 0.25/0.18 kPa/l/s).

The breathing circuit provided ports for continuous recordings of end-tidal CO_2_ pressure (PET_CO2_) and inspiratory mouth pressure (P_I_). Ports were connected with an MRI compatible pneumotachograph (ZAN 600 unit, ZAN Messgeräte GmbH, Oberhulba, Germany). A Y-valve with open expiratory port prevented re-breathing of CO_2_ while a 2.6 m tube attached to the inspiratory port allowed for the easy introduction and removal of resistive loads in the scanner environment. PET_CO2_, P_I_, tidal volume (V_T_), breathing frequency (f), minute ventilation (V_E_), and inspiratory time (T_I_) were continuously measured with the ZAN unit.

### fMRI Data Acquisition

Imaging was performed on a 3-Tesla TRIO-Magnetom Scanner (Siemens, Medical Solutions, Erlangen, Germany) using a standard 32-channel head-coil. For each data volume we acquired 48 continuous axial-slices in descending order with 2 mm × 2 mm in-plane resolution, 2 mm slice thickness and a 1 mm gap using T2^*^-weighted echoplanar imaging (TR = 2870 ms, TE = 25 ms, flip angle = 80°, field of view = 208 mm × 208 mm). The first five volumes were discarded to allow for T1-saturation. Following the experiment we also acquired a high-resolution T1-weighted structural brain scan using a standard MP-RAGE sequence (1 mm × 1 mm × 1 mm spatial resolution, 240 slices). It took subjects 13–18 min to complete the paradigm, depending on the time subjects required to complete the ratings. Consequently, the number of volumes acquired varied between 275 and 374.

### Experimental Protocol

Before starting the experiment in the scanner, subjects learned the association of visual cues and experimental conditions (see below) using standardized computer based instructions and practiced navigation through the Borg-scales on dyspnea intensity and unpleasantness. The subjects then entered the scanner with the face mask tightly fitted. A mirror attached to the head coil allowed the subjects to see the cues and scales that were projected into the bore. Before image acquisition started, subjects were allowed to familiarize with the scanner environment and the MRI-compatible button-box response-system. A test-run ensured the full visibility of all cues and scales and the tight fitting of the mask.

During the experiment, 10 blocks of mildly loaded breathing (“baseline”) alternated with 10 blocks of severely loaded breathing (“dyspnea”) using the individually pre-selected loads. Each block was visually cued for 6 s by a thin cross (red indicating baseline, green indicating dyspnea). After 6 s the thin cross changed into a solid cross and the load was introduced for 24 s. Each block of loaded breathing was followed by ratings on two Borg-scales, presented in random order: one for the unpleasantness of dyspnea and one for the intensity of dyspnea as perceived during the preceding block.

All experimental events were presented and logged using Presentation software (Neurobehavioral Systems, Inc., Albany, CA, USA). The ZAN-system, collecting the respiratory data, received triggers for the beginning of each experimental event.

### Data Analysis

Ratings for dyspnea intensity and dyspnea unpleasantness were averaged across the five early and across the five late blocks of dyspnea, respectively. The development of the ratings over time was expressed as delta (Δ) by subtracting the average across early ratings from the average across late ratings. Thus, a positive Δ indicated increasing ratings over time, interpreted as “sensitization” while a negative Δ indicated decreased ratings, interpreted as “habituation.” Δ intensity and Δ unpleasantness were tested for correlations with questionnaire data. These statistical analyzes were calculated using SPSS 20.0 software (SPSS Inc., Chicago, IL, USA).

Preprocessing and statistical analysis of fMRI data were carried out using SPM8 software^1^. Data were unwarped and realigned to the first image using six affine spatial transformation parameters, then normalized to the SPM standard template and finally smoothed using a 12 mm × 12 mm × 12 mm full-width at half-maximum Gaussian filter. Data were further filtered with a temporal highpass cut-off of 128 s. Statistical analysis on the first level was carried out within the framework of a general linear model using separate regressors for cue baseline, baseline, cue dyspnea, dyspnea, and ratings. The mean BOLD signal intensity of each volume and PET_CO2_ time logged to the beginning of each scan were included as covariates-of-no-interest. On the first level, we contrasted severe late dyspnea (blocks 6–10) with severe early dyspnea (blocks 1–5) and late cue dyspnea conditions with the early cue dyspnea conditions using the respective mild conditions as baseline. The second level analysis correlated the beta-values of the two contrast images obtained on the first level [(Δ cue dyspnea vs. cue baseline) and (Δ dyspnea vs. baseline)] with Δ intensity and Δ unpleasantness using separate models. All models included individual Δ breathing frequency and Δ inspiratory mouth pressure (averaged across cue dyspnea vs. cue baseline and dyspnea vs. baseline conditions, respectively) as covariates-of-no-interest as these breathing parameters showed slight, but statistically significant changes over time (see Results).

Correlations throughout the brain were accepted as significant if exceeding a family-wise-error (FWE) corrected threshold of *p* < 0.05. Given our *a priori* hypotheses for habituation/sensitization-related correlations during dyspnea anticipation und perception we conducted further region-of interest (ROI) analyses. Masks for the insula, the amygdala, the ACC, OFC, and dmPFC were generated from the automated anatomical labeling (AAL) template described by Tzourio-Mazoyer et al. (2002). A midbrain ROI centered on PAG was defined using a 10 mm sphere around the average coordinates for PAG activation reported by Linnman et al. (2012). Activation within these ROIs was considered significant, if exceeding as threshold of *p* < 0.05, corrected for multiple comparisons within each ROI.

An additional ROI covering the visual cortex was included as control area. Changes in brain activation within this area were not expected to show any significant correlation with changes in dyspnea intensity and unpleasantness ratings over time. For this ROI we employed a more liberal threshold of *p* < 0.001 uncorrected.

## Results

### Ratings

**FIGURE 1:**
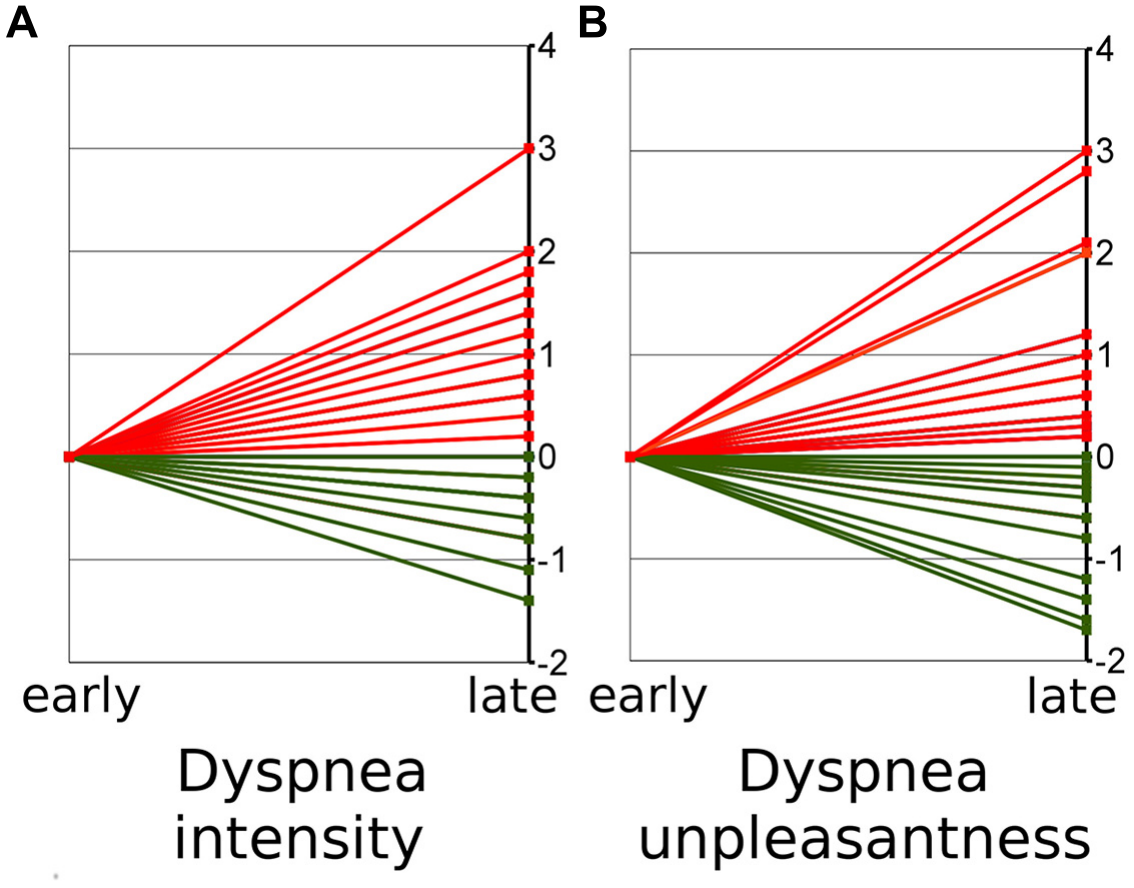
**Early (mean of block 1–5) and late (mean of block 6–10) ratings on dyspnea intensity (A) and unpleasantness (B)**. The figure shows the change from early ratings (mean set to zero) to late ratings. Red lines thus indicate increases of ratings over time (“sensitization”) while green lines indicate decreases (“habituation”).

In 30.5% of all subjects we observed decreasing ratings over time indicating habituation for both dyspnea intensity and dyspnea unpleasantness. In 54.5% we observed increasing ratings with regard to dyspnea intensity, while 63% showed increasing dyspnea unpleasantness ratings indicating sensitization. In the remainder of subjects (seven and three subjects, respectively) Δ intensity and Δ unpleasantness equaled zero (**Table 1**). The Δ for dyspnea intensity ranged from -1.3 to +3 while Δ unpleasantness ranged from -1.7 to +3 (**Figure 1**, see Supplementary Figure S1 for absolute ratings).

**Table 1:** Portion of subjects (%) showing sensitization, habituation, and no changes for Δ intensity and Δ unpleasantness.

	Sensitization	No change	Habituation
Δ intensity	25 (54.5%)	7 (15%)	14 (30.5%)
Δ unpleasantness	29 (63%)	3 (6.5%)	14 (30.5%)

### Personality Traits

**FIGURE 2:**
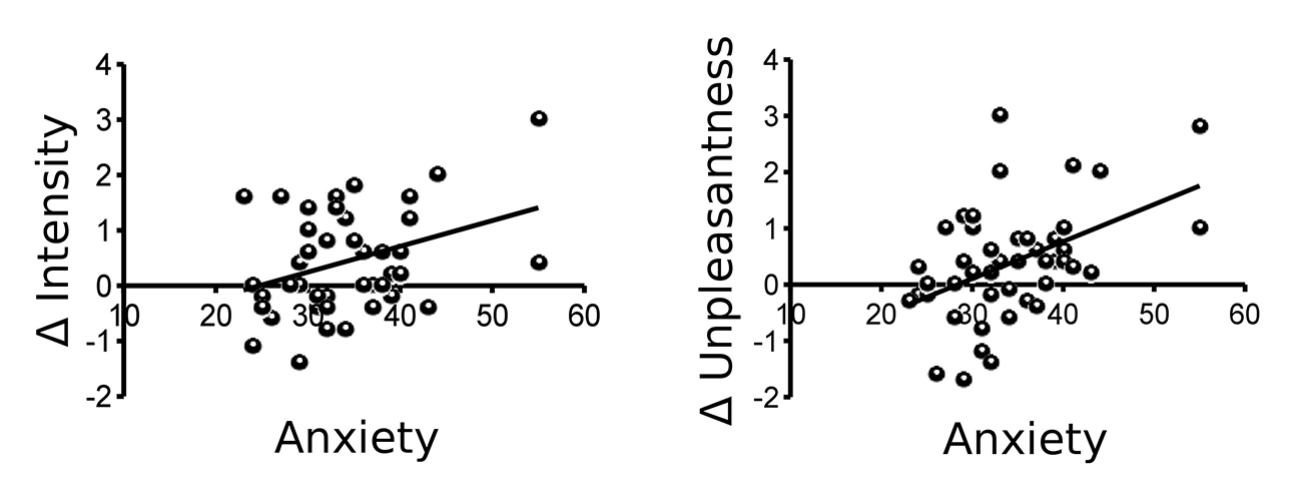
**Trait anxiety as measured by the State-Trait-Anxiety Inventory (STAI-T) correlates significantly with both Δ intensity (*r* = 0.36, *p* = 0.007) and Δ unpleasantness (*r* = 0.46, *p* = 0.001) of dyspnea ratings (mean late-mean early)**.

Both, state and trait anxiety as measured using the STAI-T and STAI-S questionnaires, showed a significant positive correlation with Δ unpleasantness (both *r* = 0.46, *p* = 0.001) and Δ intensity (*r* = 0.36 and 0.32, respectively, *p* = 0.007 and 0.017, respectively). As higher scores indicate higher anxiety levels, a positive correlation indicates that individuals with higher anxiety were more likely to show sensitization while subjects with low anxiety were more likely to show habituation (**Figure 2**). When early and late ratings instead of difference scores were correlated with anxiety scores, only late dyspnea intensity and unpleasantness ratings showed a significant relation with anxiety (Supplementary Figure S2).

### Respiratory Parameters

The changes of respiratory parameters between dyspnea and baseline blocks were comparable for late vs. early blocks with the exception of ΔPET_CO2_, Δf, and ΔP_I_ (**Table 2**). The relation between PET_CO2_ during dyspnea as compared to baseline showed a slight but significant increase over time, while P_I_ decreased slightly over time during dyspnea as compared to baseline. The difference of f between cue dyspnea and cue baseline decreased from early to late blocks. While fluctuations in PET_CO2_ were accounted for on the first level analysis, fluctuations of P_I_ and f were included into the second level analysis as covariates-of-no-interest (see Materials and Methods).

**Table 2:** Mean (SD) Δ of breathing parameters between early and late experimental blocks (Δ cue dyspnea vs. cue baseline and Δ dyspnea vs. baseline averaged across subjects).

	Mean (SD) cue dyspnea vs. cue baseline	Mean (SD) dyspnea vs. baseline
Δ PET_CO2_ (mmHG)	0.08 (1.34)	0.27 (0.56)^*^
Δ V_T_ (L)	0.004 (0.18)	0.03 (0.16)
Δ V_E_ (L/min)	-0.03 (1.57)	0.09 (1.32)
Δ T_I_ (s)	0.08 (0.46)	0.1 (0.29)
Δ f (breaths/min)	-0.88 (1.71)^*^	-0.31 (0.9)
Δ P_I_ (mbar)	-0.07 (0.38)	-0.81 (1.76)^*^

^*^p < 0.05 for one-sample t-tests, corrected for multiple comparisons.

*PET_CO2_, end-tidal CO_2_ pressure; V_T_, tidal volume; V_E_, minute ventilation; T_I_, inspiratory time; f, breathing frequency; P_I_, peak inspiratory mouth pressure*.

### fMRI

There were no significant differences of either dyspnea anticipation or dyspnea perception between the first (blocks 1–5) and second (blocks 6–10) half of the experiment. The whole-brain analysis based on a FWE-corrected *p* < 0.05 showed no significant correlations of Δ intensity or Δ unpleasantness during dyspnea anticipation (Δ cue dyspnea vs. cue baseline) or dyspnea perception (Δ dyspnea vs. baseline). The visual cortex as a control area showed no significant correlations with either Δ intensity or Δ unpleasantness during either dyspnea anticipation or dyspnea perception at a liberal threshold of *p* uncorr < 0.001.

**FIGURE 3:**
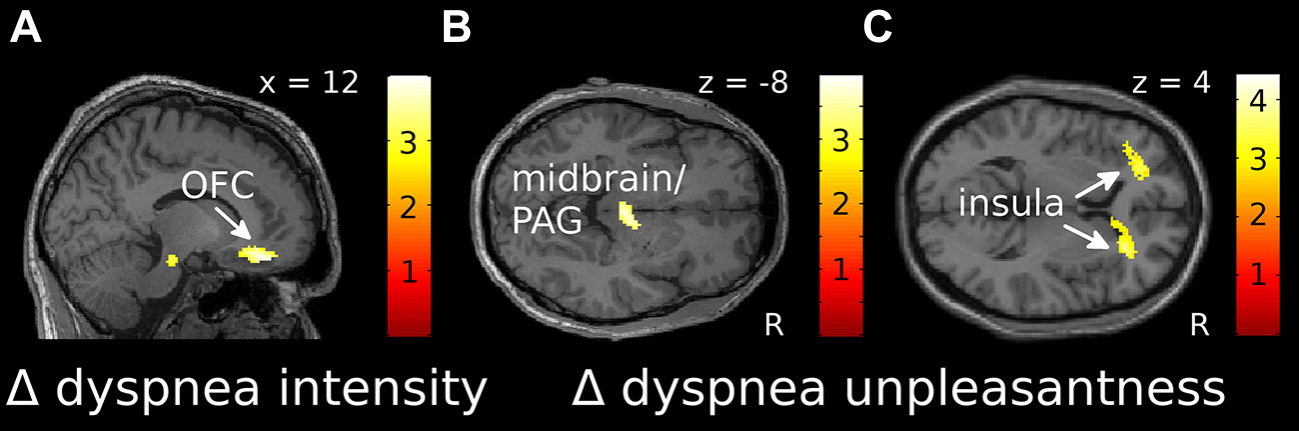
**Localization of correlations between Δ intensity (A) and Δ unpleasantness (B,C) ratings and changes in fMRI activation over time for dyspnea anticipation [(cue dyspnea late-cue baseline late)-(cue dyspnea early-cue baseline early)]. (A)** and **(B)** show negative correlations, while **(C)** shows positive correlations. Correlations displayed at *p* uncorr < 0.005 are superimposed on a representative single subjects T1-weighted MR image. Color-bars indicate *T*-values. OFC, orbitofrontal cortex; PAG, periaqueductal gray; R, right.

For dyspnea anticipation, the ROI-based analysis showed a significant negative correlation of Δ intensity with the Δ of brain activation (Δ cue dyspnea vs. cue baseline) within the right OFC, extending into the ACC (**Figure 3A**, Supplementary Figure S3A). This indicated that sensitization toward dyspnea intensity was associated with decreased OFC activation, while habituation was associated with increasing OFC activation. For Δ unpleasantness a significant negative correlation was found within the midbrain/PAG (**Figure 3B**, Supplementary Figure S3B) while positive correlations were found for the anterior insular cortex bilaterally (**Figure 3C**, Supplementary Figures S3C–E). Sensitization toward dyspnea unpleasantness was, thus, associated with decreasing activation in the midbrain/PAG and increasing activation in the anterior insula. Conversely, habituation was associated with increasing midbrain/PAG activation and decreasing anterior insula activation.

**FIGURE 4:**
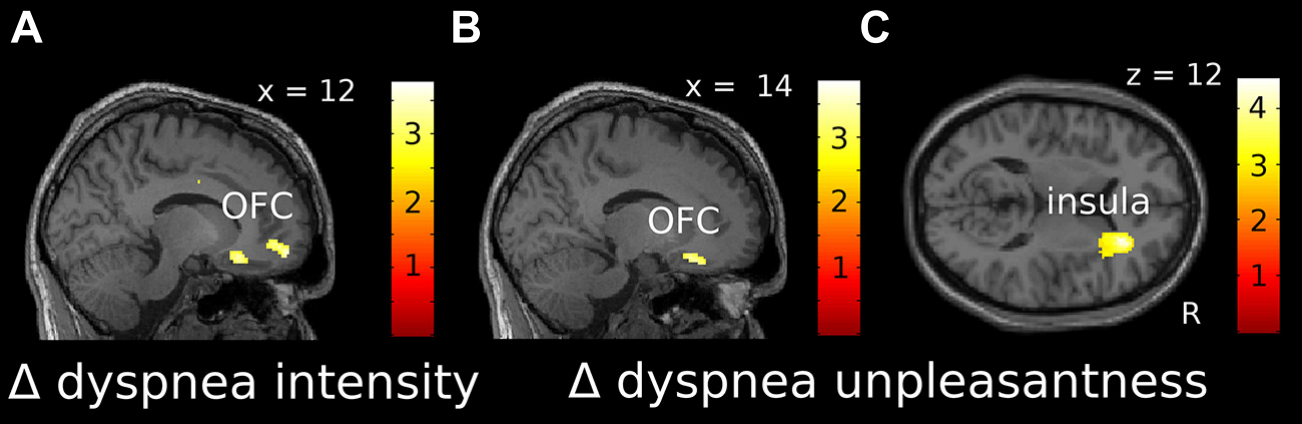
**Localization of correlations between Δ intensity (A) and Δ unpleasantness (B,C) ratings and changes in fMRI activation over time for dyspnea perception [(dyspnea late-baseline late)-(dyspnea early-baseline early)]. (A)** and **(B)** show negative correlations, while **(C)** shows positive correlations. Correlations displayed at *p* uncorr < 0.005 are superimposed on a representative single subjects T1-weighted MR image. Color-bars indicate *T*-values. OFC, orbitofrontal cortex; R, right.

For dyspnea perception (Δ dyspnea vs. baseline), we found a significant negative correlation within the right OFC for both Δ intensity and Δ unpleasantness, which extended into the ACC (**Figures 4A,B**, Supplementary Figures S3F,G). Thus, as during dyspnea anticipation, increasing OFC activation was associated with habituation, while decreasing OFC activation was associated with sensitization toward dyspnea, for both, intensity and unpleasantness. The Δ of brain activation (Δ dyspnea vs. baseline) within the right anterior insular cortex showed a significant positive correlation with Δ unpleasantness (**Figure 4C**, Supplementary Figure S3H), indicating that increasing activation of the anterior insular cortex during dyspnea perception was associated with sensitization and decreasing activation with habituation. Coordinates, *Z*-, *r*-, and *p*-values are summarized in **Table 3**.

**Table 3:** MNI-space coordinates, *Z*-, *r*-, and small-volume corrected *p*-values for peak voxels within areas showing a significant correlation with Δ intensity and Δ unpleasantness (partial correlations controlled for changes in breathing parameters *P_I_* and *f*).

		*x*	*y*	*z*	*Z*	*r*	p^*^
**Dyspnea anticipation (Δ cue dyspnea vs. cue baseline)**			
with Δ intensity	OFC R	12	42	-20	3.47	-0.5	0.04
with Δ unpleasantness	Midbrain/PAG	2	-20	-8	3.10	-0.45	0.04
	Insula R	26	32	4	3.40	0.49	0.05
	Insula L	-34	22	14	3.87	0.55	0.01
		-36	34	8	3.47	0.5	0.04
**Dyspnea Perception (Δ dyspnea vs. baseline)**			
with Δ intensity	OFC R	14	56	-14	3.34	-0.47	0.05
with Δ unpleasantness	OFC R	16	30	-20	3.32	-0.46	0.05
	Insula R	26	30	12	4.01	0.57	0.01

^*^Corrected for multiple comparisons in respective ROI.

*MNI, Montreal Neurological Institute; OFC, orbitofrontal cortex; PAG, periaqueductal gray; R, right, L, left*.

To investigate whether brain activation changes were rather related to general anxiety than changes in dyspnea intensity or unpleasantness perception, we included individual STAI-T scores as additional covariate-of -no-interest in a *post hoc* analysis, correlating extracted beta-values from the brain areas reported in **Table 3** with Δ intensity and Δ unpleasantness, respectively, (Supplementary Table S1). Correlations within all areas maintained significant in this analysis. When STAI-T scores were directly correlated with the extracted beta-values, mainly cue-related beta-values showed significant correlations with general anxiety. These correlations were attenuated or disappeared when changes in breathing parameters and dyspnea ratings were added as control variables (Supplementary Table S2).

## Discussion

In this study we investigated the development of perceived dyspnea intensity and unpleasantness over repeated blocks of dyspnea together with parallel changes of brain activation. We observed significant correlations of late vs. early dyspnea ratings (Δ intensity and Δ unpleasantness) with late vs. early brain activity during dyspnea anticipation and dyspnea perception. These correlations were restricted to a subset of our ROIs, namely the OFC, midbrain/PAG, and the anterior insular cortex. A control area covering the visual cortex failed to show any significant rating-related changes indicating the specificity of our findings. While brain activity changes in the OFC and the midbrain/PAG were negatively correlated with Δ intensity and/or Δ unpleasantness, respectively, the anterior insular cortex showed positive correlations. Correlations were furthermore specific to the dimension of perceived dyspnea: Only Δ unpleasantness ratings showed significant positive correlations with the anterior insular cortex, and a negative correlation with the midbrain/PAG. While negative correlations of brain activation changes in the midbrain/PAG with Δ unpleasantness were limited to the anticipation period, the significant correlations of the OFC and the anterior insula were observed for both, dyspnea anticipation and dyspnea perception.

Both, Δ intensity and Δ unpleasantness, were significantly correlated with state and trait anxiety levels. This is in line with the notion that high anxious as compared to low anxious individuals are less likely to show short-term habituation toward dyspnea, which has previously been shown for hypercapnic rebreathing (Li et al., 2006). In addition, data from Wan et al. (2008, 2012) suggest that the effect of anxiety on short-term habituation is more pronounced for dyspnea unpleasantness as compared to dyspnea intensity. This is partly supported by our data, as correlations for state and trait anxiety with Δ unpleasantness were more pronounced (*r* = 0.46) compared to correlations with Δ intensity (0.32 and 0.36, respectively). Moreover, while the portion of individuals showing habituation toward dyspnea was identical for the two dimensions of dyspnea perception, the portion of subjects showing sensitization was higher for dyspnea unpleasantness (63%) as compared to dyspnea intensity (54.5%).

The close relationship of Δ intensity and Δ unpleasantness with anxiety raises the question, whether any correlation of changes in brain activity might be better explained by individual anxiety scores rather than rating dynamics. However, *post hoc* partial correlation analyses yielded no support for this notion.

The functional neuroimaging data presented here mirror the higher impact of trial repetition on dyspnea unpleasantness as compared to dyspnea intensity. While significant correlations of Δ intensity with changes in brain activation were limited to the OFC, Δ unpleasantness was significantly correlated with changes in the midbrain/PAG and the bilateral anterior insular cortex as well. This observation of short-term habituation/sensitization in healthy individuals parallels results from a previous study in patients with asthma, which linked long-term habituation toward dyspnea unpleasantness with similar brain areas (von Leupoldt et al., 2009). More specifically, that study observed reduced insula activation and increased PAG activation to resistive load induced dyspnea in asthma patients compared to healthy controls, which were paralleled by reduced dyspnea unpleasantness ratings in the patient group and correlated with asthma duration (von Leupoldt et al., 2009). Interestingly, longer asthma duration and reduced dyspnea unpleasantness in the same patients were also correlated with structural brain changes in terms of increased gray matter volume in the PAG, which was interpreted as another potential mechanism of long-term habituation to dyspnea (von Leupoldt et al., 2011b).

The PAG, in particular, is thought to be a key area of the so called anti-nociceptive network as repeatedly shown by studies on pain modulation (e.g., Fairhurst et al., 2007; for review see Tracey and Mantyh, 2007). Interestingly, midbrain/PAG activation during pain anticipation has been demonstrated to have a significant effect on subsequent pain perception (Brodersen et al., 2012). This is in line with our results, showing a significant correlation of Δ unpleasantness with midbrain/PAG activation changes for dyspnea anticipation only. For the OFC, there was a significant effect on pain perception for both, brain activation during pain anticipation and during painful simulation (Brodersen et al., 2012). This is also in line with the data presented here, as a significant correlation of changes in dyspnea ratings with changes in brain activation was found during anticipation and perception of dyspnea. Increasing activation in both areas, midbrain/PAG and OFC, were related to decreasing ratings of dyspnea unpleasantness indicating that increased activation in these areas support habituation. Further studies are required to investigate whether habituation is effectuated, e.g., by top–down (OFC) and/or bottom–up (midbrain/PAG) inhibition of other brain areas relevant for dyspnea perception (von Leupoldt and Dahme, 2005; Davenport and Vovk, 2009; Evans, 2010). However, one candidate target area for inhibition is the insular cortex, as this area showed the reversed correlation pattern: increasing activation in the insular cortex was related to increasing ratings of dyspnea unpleasantness. A similar pattern of insular and midbrain/PAG activation was described for the anticipation of pain by Ploner et al. (2010).

Changes of anterior insular activation over time were significantly linked to changes in dyspnea unpleasantness only. Therefore, the present findings support the notion, that activation of the anterior insular cortex is particularly relevant for the perception of the affective dimension of aversive events in general (Seeley et al., 2007) and dyspnea unpleasantness in particular (von Leupoldt et al., 2008; Paulus et al., 2012).

The positive correlations of changes in dyspnea ratings with brain activation changes during the preceding anticipation periods suggest an effect of expectancy and/or learning. As our results are limited to short-term habituation/sensitization in healthy individuals, future studies on long-term habituation and sensitization in patients with chronic dyspnea are required to investigate whether this association between anticipatory brain activations and subsequent dyspnea experience provides a potential target to correct for unfavorable learning of either avoidance behavior (maladaptive sensitization) or under-medication (maladaptive habituation).

In this study we present a novel approach for the investigation of habituation and sensitization toward dyspnea by using the correlation between late vs. early ratings on perceived dyspnea and late vs. early brain activation patterns. This approach allowed the inclusion of all participants irrespective of individual rating patterns as the variance across subjects allowed to differentiate between habituation and sensitization processes. However, correlation studies are limited in their ability to derive causal or directional conclusions. The investigation of connectivity patterns between the brain areas identified as critical in this study and by others might be a useful next step to clarify the way in which relevant areas interact and modulate each other. The connection between state and trait anxiety and habituation/sensitization toward dyspnea suggested by others (Li et al., 2006; Wan et al., 2008, 2012) and confirmed by this study provides another promising target for future studies on treatment and rehabilitation optimization.

## Author Contributions

MCS: Substantial contributions to the design of the work, data acquisition, data analysis, drafting the manuscript and revising it critically for important intellectual content, final approval of the version to be published, agreement to be accountable for all aspects of the work in ensuring that questions related to the accuracy or integrity of any part of the work are appropriately investigated and resolved.

RE: Substantial contributions to the design of the work, data acquisition, revising the manuscript critically for important intellectual content, final approval of the version to be published, agreement to be accountable for all aspects of the work in ensuring that questions related to the accuracy or integrity of any part of the work are appropriately investigated and resolved.

MG: Substantial contributions to the design of the work, interpretation of data for the work, data analysis, revising the manuscript critically for important intellectual content, final approval of the version to be published, agreement to be accountable for all aspects of the work in ensuring that questions related to the accuracy or integrity of any part of the work are appropriately investigated and resolved.

CB: Substantial contributions to the design of the work, interpretation of data for the work, revising the manuscript critically for important intellectual content, final approval of the version to be published, agreement to be accountable for all aspects of the work in ensuring that questions related to the accuracy or integrity of any part of the work are appropriately investigated and resolved.

AvL: Substantial contributions to the conception and design of the work, interpretation of data for the work, revising the manuscript critically for important intellectual content, final approval of the version to be published, agreement to be accountable for all aspects of the work in ensuring that questions related to the accuracy or integrity of any part of the work are appropriately investigated and resolved.

## Conflict of Interest Statement

The authors declare that the research was conducted in the absence of any commercial or financial relationships that could be construed as a potential conflict of interest.

## Footnotes

1 www.fil.ion.ucl.ac.uk/spm

## References

[B1] Bingel U. Schoell E. Herken W. Büchel C. May A. (2007). Habituation to painful stimulation involves the antinociceptive system. *Pain* 131 21–30. 10.1016/j.pain.2006.12.005 17258858

[B2] Bloch-Salisbury E. Shea S. A. Brown R. Evans K. Banzett R. B. (1996). Air hunger induced by acute increase in PCO2 adapts to chronic elevation of PCO2 in ventilated humans. *J. Appl. Physiol.* 81 949–956. 8872667 10.1152/jappl.1996.81.2.949

[B3] Brodersen K. H. Wiech K. Lomakina E. I. Lin C.-S. Buhmann J. M. Bingel U. (2012). Decoding the perception of pain from fMRI using multivariate pattern analysis. *Neuroimage* 63 1162–1170. 10.1016/j.neuroimage.2012.08.035 22922369 PMC3532598

[B4] Carrieri-Kohlman V. Gormley J. M. Eiser S. Demir-Deviren S. Nguyen H. Paul S. M. (2001). Dyspnea and the affective response during exercise training in obstructive pulmonary disease. *Nurs. Res.* 50 136–146. 10.1097/00006199-200105000-00002 11393635

[B5] Davenport P. W. Vovk A. (2009). Cortical and subcortical central neural pathways in respiratory sensations. *Respir. Physiol. Neurobiol.* 167 72–86. 10.1016/j.resp.2008.10.001 18977463

[B6] Evans K. C. (2010). Cortico-limbic circuitry and the airways: insights from functional neuroimaging of respiratory afferents and efferents. *Biol. Psychol.* 84 13–25. 10.1016/j.biopsycho.2010.02.005 20211221 PMC2908728

[B7] Fairhurst M. Wiech K. Dunckley P. Tracey I. (2007). Anticipatory brainstem activity predicts neural processing of pain in humans. *Pain* 128 101–110. 10.1016/j.pain.2006.09.001 17070996

[B8] Hayen A. Herigstad M. Pattinson K. T. S. (2013). Understanding dyspnea as a complex individual experience. *Maturitas* 76 45–50. 10.1016/j.maturitas.2013.06.005 23849705

[B9] Hayen A. Herigstad M. Wiech K. Pattinson K. T. S. (2015). Subjective evaluation of experimental dyspnoea – Effects of isocapnia and repeated exposure. *Respir. Physiol. Neurobiol.* 208 21–28. 10.1016/j.resp.2014.12.019 25578628 PMC4347539

[B10] Herigstad M. Hayen A. Wiech K. Pattinson K. T. S. (2011). Dyspnoea and the brain. *Respir. Med.* 105 809–817. 10.1016/j.rmed.2010.12.022 21295457

[B11] Holtz K. Pané-Farré C. A. Wendt J. Lotze M. Hamm A. O. (2012). Brain activation during anticipation of interoceptive threat. *Neuroimage* 61 857–865. 10.1016/j.neuroimage.2012.03.019 22440646

[B12] Hsieh J. C. Stone-Elander S. Ingvar M. (1999). Anticipatory coping of pain expressed in the human anterior cingulate cortex: a positron emission tomography study. *Neurosci. Lett.* 262 61–64. 10.1016/S0304-3940(99)00060-9 10076873

[B13] Janssens T. Verleden G. De Peuter S. Van Diest I. Van den Bergh O. (2009). Inaccurate perception of asthma symptoms: a cognitive-affective framework and implications for asthma treatment. *Clin. Psychol. Rev.* 29 317–327. 10.1016/j.cpr.2009.02.006 19285771

[B14] Labus J. S. Naliboff B. D. Berman S. M. Suyenobu B. Vianna E. P. Tillisch K. (2009). Brain networks underlying perceptual habituation to repeated aversive visceralstimuli in patients with iritable bowel syndrome. *Neuroimage* 47 952–960. 10.1016/j.neuroimage.2009.05.078 19501173 PMC3399695

[B15] Lansing R. W. Gracely R. H. Banzett R. B. (2009). The multiple dimensions of dyspnea: review and hypotheses. *Respir. Physiol. Neurobiol.* 167 53–60. 10.1016/j.resp.2008.07.012 18706531 PMC2763422

[B16] Laviolette L. Laveneziana P. ERS Research Seminar Faculty. (2014). Dyspnoea: a multidimensional and multidisciplinary approach. *Eur. Respir. J.* 43 1750–1762. 10.1183/09031936.00092613 24525437

[B17] Li W. Daems E. Van de Woestijne K. P. Van Diest I. Gallego J. De Peuter S. (2006). Air hunger and ventilation in response to hypercapnia: effects of repetition and anxiety. *Physiol. Behav.* 88 47–54. 10.1016/j.physbeh.2006.03.001 16626764

[B18] Linnman C. Moulton E. A. Barmettler G. Becerra L. Borsook D. (2012). Neuroimaging of the periaqueductal gray: state of the field. *Neuroimage* 60 505–522. 10.1016/j.neuroimage.2011.11.095 22197740 PMC3288184

[B19] Lowén M. B. O. Mayer E. Tillisch K. Labus J. Naliboff B. Lundberg P. (2015). Deficient habituation to repeated rectal distensions in irritable bowel syndrome patients with visceral hypersensitivity. *Neurogastroenterol. Motil.* 27 646–655. 10.1111/nmo.12537 25777251

[B20] Miller M. R. Hankinson J. Brusasco V. Burgos F. Casaburi R. Coates A. (2005). Standardisation of spirometry. *Eur. Respir. J.* 26 319–338. 10.1183/09031936.05.00034805 16055882

[B21] Parshall M. B. Schwartzstein R. M. Adams L. Banzett R. B. Manning H. L. Bourbeau J. (2012). An official American Thoracic Society statement: update on the mechanisms, assessment, and management of dyspnea. *Am. J. Respir. Crit. Care Med.* 185 435–452. 10.1164/rccm.201111-2042ST 22336677 PMC5448624

[B22] Paulus M. P. Flagan T. Simmons A. N. Gillis K. Kotturi S. Thom N. (2012). Subjecting elite athletes to inspiratory breathing load reveals behavioral and neural signatures of optimal performers in extreme environments. *PLoS ONE* 7:e29394 10.1371/journal.pone.0029394 PMC3261851 22276111

[B23] Petersen S. Schroijen M. Mölders C. Zenker S. Van den Bergh O. (2014). Categorical interoception: perceptual organization of sensations from inside. *Psychol. Sci.* 25 1059–1066. 10.1177/0956797613519110 24570260

[B24] Petersen S. van den Berg R. A. Janssens T. Van den Bergh O. (2011). Illness and symptom perception: a theoretical approach towards an integrative measurement model. *Clin. Psychol. Rev.* 31 428–439. 10.1016/j.cpr.2010.11.002 21146271

[B25] Petrovic P. Kalso E. Petersson K. M. Ingvar M. (2002). Placebo and opioid analgesia– imaging a shared neuronal network. *Science* 295 1737–1740. 10.1126/science.1067176 11834781

[B26] Ploner M. Lee M. C. Wiech K. Bingel U. Tracey I. (2010). Prestimulus functional connectivity determines pain perception in humans. *Proc. Natl. Acad. Sci. U.S.A.* 1071 355–360. 10.1073/pnas.0906186106 19948949 PMC2806712

[B27] Seeley W. W. Menon V. Schatzberg A. F. Keller J. Glover G. H. Kenna H. (2007). Dissociable intrinsic connectivity networks for salience processing and executive control. *J. Neurosci.* 279 2349–2356. 10.1523/JNEUROSCI.5587-06.2007 17329432 PMC2680293

[B28] Spielberger C. D. Gorsuch R. L. Lushene R. Vagg P. R. Jacobs G. A. (1983). *Manual for the State-Trait Anxiety Inventory*. Palo Alto, CA: Consulting Psychologists Press.

[B29] Stankewitz A. Valet M. Schulz E. Wöller A. Sprenger T. Vogel D. (2013). Pain sensitisers exhibit grey matter changes after repetitive pain exposure: a longitudinal voxel-based morphometry study. *Pain* 154 1732–1737. 10.1016/j.pain.2013.05.019 23685021

[B30] Tracey I. Mantyh P. W. (2007). The cerebral signature for pain perception and its modulation. *Neuron* 55 377–391. 10.1016/j.neuron.2007.07.012 17678852

[B31] Troosters T. van der Molen T. Polkey M. Rabinovich R. A. Vogiatzis I. Weisman I. (2013). Improving physical activity in COPD: towards a new paradigm. *Respir. Res.* 14 115 10.1186/1465-9921-14-115 PMC4176094 24229341

[B32] Tzourio-Mazoyer N. Landeau B. Papathanassiou D. Crivello F. Etard O. Delcroix N. (2002). Automated anatomical labeling of activations in SPM using a macroscopic anatomical parcellation of the MNI MRI single-subject brain. *Neuroimage* 15 273–289. 10.1006/nimg.2001.0978 11771995

[B33] von Leupoldt A. Dahme B. (2005). Cortical substrates for the perception of dyspnea. *Chest* 128 345–354. 10.1378/chest.128.1.345 16002956

[B34] von Leupoldt A. Sommer T. Kegat S. Baumann H. J. Klose H. Dahme B. (2008). The unpleasantness of perceived dyspnea is processed in the anterior insula and amygdala. *Am. J. Respir. Crit. Care Med.* 177 1026–1032. 10.1164/rccm.200712-1821OC 18263796

[B35] von Leupoldt A. Sommer T. Kegat S. Eippert F. Baumann H. J. Klose H. (2009). Down-regulation of insular cortex responses to dyspnea and pain in asthma. *Am. J. Respir. Crit. Care Med.* 180 232–238. 10.1164/rccm.200902-0300OC 19483110

[B36] von Leupoldt A. Vovk A. Bradley M. M. Lang P. J. Davenport P. W. (2011a). Habituation in neural processing and subjective perception of respiratory sensations. *Psychophysiology* 48 808–812. 10.1111/j.1469-8986.2010.01141.x 21039587 PMC3627367

[B37] von Leupoldt A. Brassen S. Baumann H. J. Klose H. Büchel C. (2011b). Structural brain changes related to disease duration in patients with asthma. *PLoS ONE* 6:e23739 10.1371/journal.pone.0023739 PMC3158798 21886820

[B38] Wan L. Stans L. Bogaerts K. Decramer M. Van den Bergh O. (2012). Sensitization in medically unexplained dyspnea: differential effects on intensity and unpleasantness. *Chest* 141 989–995. 10.1378/chest.11-1423 22016486

[B39] Wan L. Van Diest I. De Peuter S. Bogaerts K. Oyen N. Hombroux N. (2008). Repeated experiences of air hunger and ventilatory behavior in response to hypercapnia in the standardized rebreathing test: effects of anxiety. *Biol. Psychol.* 77 223–232. 10.1016/j.biopsycho.2007.10.013 18077078

[B40] Wan L. Van Diest I. De Peuter S. Bogaerts K. Van den Bergh O. (2009). Repeated breathlessness experiences induced by hypercapnia: differential effects on intensity and unpleasantness. *Chest* 135 455–461. 10.1378/chest.08-1226 19201712

