# Supplementary material for: Brain mechanisms of short-term habituation and sensitization toward dyspnea
Source: Front Psychol. 2015 Jun 2;6:748. doi: 10.3389/fpsyg.2015.00748 (PMC4451234; doi:10.3389/fpsyg.2015.00748)
Supplement: Supplementary file 1 [file Data_Sheet_1.pdf]

Supplementary Material

Brain mechanisms of short-term habituation and sensitization towards dyspnea

M Cornelia Stoeckel 1, Roland W Esser 1, Matthias Gamer 1, Christian Büchel 1, Andreas von Leupoldt 1,2*

1 Department of Systems Neuroscience, University Medical Center Hamburg-Eppendorf, Hamburg, Germany

2 Research Group Health Psychology, University of Leuven, Leuven, Belgium

***Correspondence**

Prof. Dr. Andreas von Leupoldt

Research Group Health Psychology

University of Leuven

Tiensestraat 102

3000 Leuven, Belgium

Email: andreas.vonleupoldt@ppw.kuleuven.be

Supplementary Figures

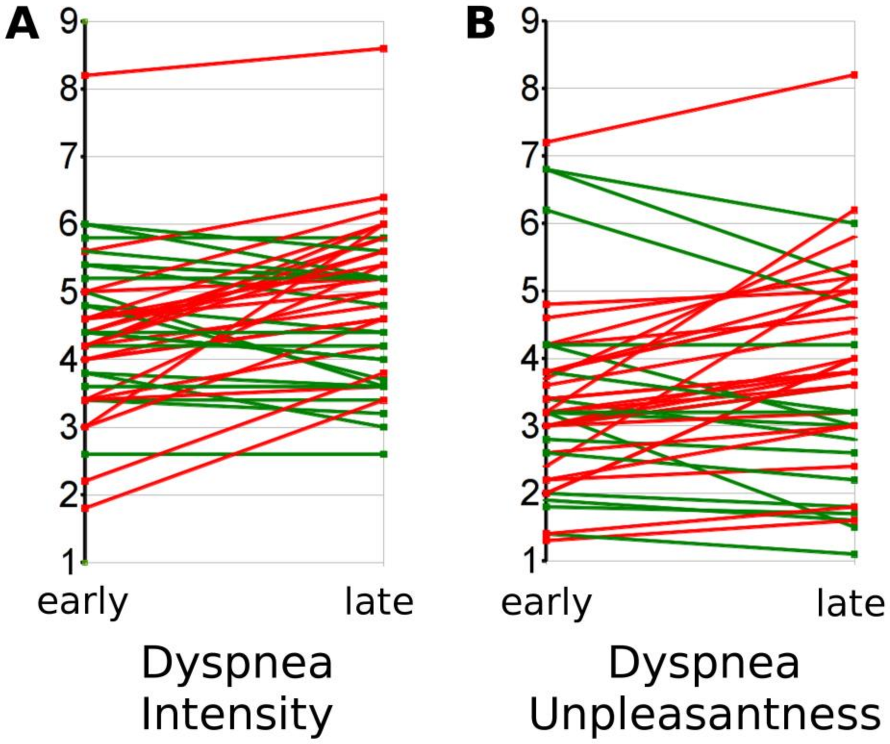

Supplementary Figure 1. Individual early (mean of block 1-5) and late (mean of block 6-10) ratings on dyspnea intensity (**A**) and unpleasantness (**B**). Increases in ratings are highlighted in red while unchanged and decreased ratings are highlighted in green.

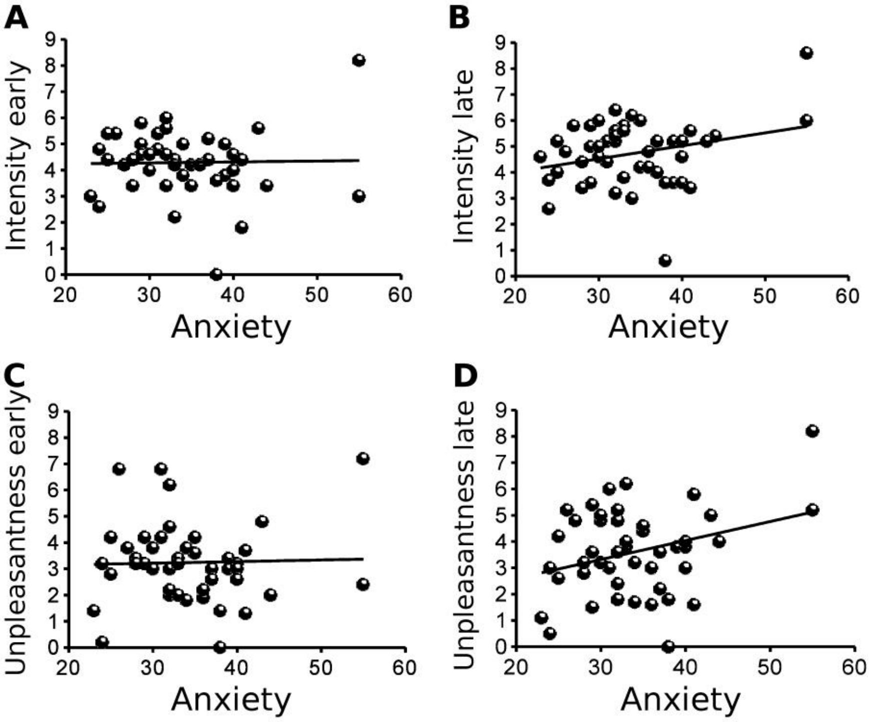

Supplementary Fig. 2 Correlations of trait anxiety as measured by the STAI-T with early (averaged across blocks 1-5) and late (averaged across blocks 6-10) intensity and unpleasantness ratings. Trait anxiety shows no significant correlation with early intensity (**A**) or unpleasantness (**C**) ratings ($r = 0.02$, $p = 0.45$ and $r = 0.03$, $p = 0.43$, respectively). However, during the second half of the experiment, both intensity (**B**) and unpleasantness (**D**) ratings are significantly correlated with trait anxiety ($r = 0.28$, $p = 0.03$ and $r = 0.31$, $p = 0.02$, respectively).

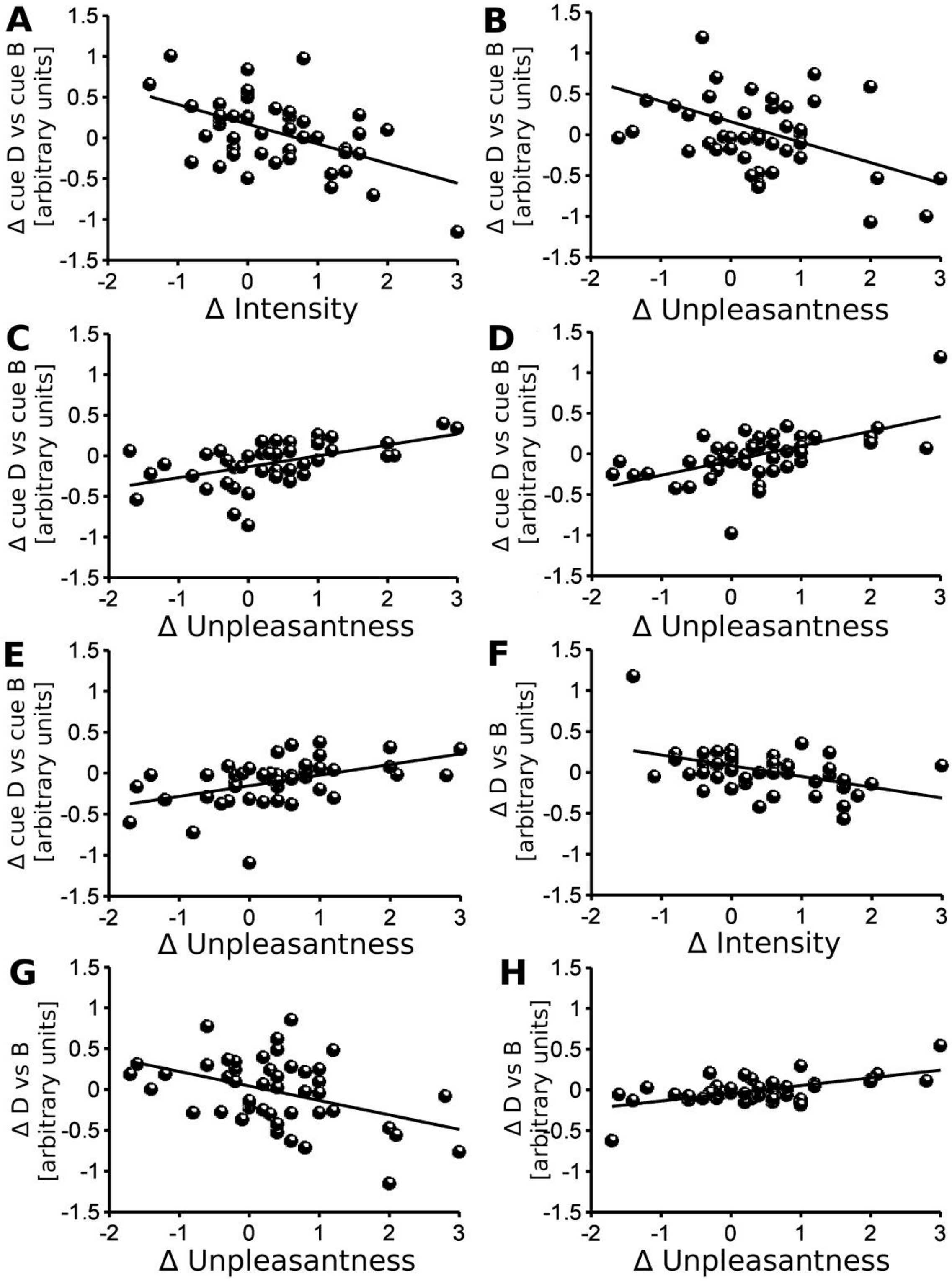

Supplementary Figure 3. Significant bivariate correlations of late (block 6-10) vs early (blocks 1-5) intensity and unpleasantness ratings with late vs early brain activations during either dyspnea anticipation (**A-E**) or dyspnea perception (**F-H**).

(**A**) Significant negative correlation of Δ intensity with changes in anticipatory brain activation [Δ cue dyspnea vs. cue baseline] within the right orbitofrontal cortex (x = 12, y = 42, z = -20, r = -0.51, p < 0.001). (**B**) Significant negative correlation of Δ unpleasantness with changes in anticipatory brain activation [Δ cue dyspnea vs. cue baseline] within the midbrain/PAG (x = 2, y = -20, z = -8, r = -0.46, p = 0.001). (**C**) Significant positive correlation of Δ unpleasantness with changes in anticipatory brain activation [Δ cue dyspnea vs. cue baseline] within the right anterior insular cortex (x = 26, y = 32, z = 4, r = 0.52, p < 0.001). (**D**) Significant positive correlation of Δ unpleasantness with changes in anticipatory brain activation [Δ cue dyspnea vs. cue baseline] within the left insular cortex (x = -34, y = 22, z = 14, r = 0.58, p < 0.001). (**E**) Significant positive correlation of Δ unpleasantnes with changes in anticipatory brain activation [Δ cue dyspnea vs. cue baseline] within the left anterior insular cortex (x = -36, y = 34, z = 8, r = 0.47, p = 0.001). (**F**) Significant negative correlation of Δ intensity with changes in dyspnea related brain activation [Δ dyspnea vs. baseline] within the right orbitofrontal cortex (x = 14, y = 56, z = -14, r = -0.44, p = 0.001). (**G**) Significant negative correlation of Δ unpleasantness with changes in anticipatory brain activation [Δ dyspnea vs. baseline] within the right orbitofrontal cortex (x = 16, y = 30, z = -20, r = -0.43, p = 0.002). (**H**) Significant positive correlation of Δ unpleasantness with changes in anticipatory brain activation [Δ dyspnea vs. baseline] within the right anterior insular cortex (x = 26, y = 30, z = 12, r = 0.58, p < 0.001).

Supplementary Tables

Supplementary Table 1. When the correlations between changes in brain activation from early (blocks 1-5) to late blocks (block 6-10) with changes in dyspnea intensity and unpleasantness ratings previously only controlled for changes in breathing parameters (P_I and f) are additionally controlled for trait-anxiety (as measured using the STAI-T), correlations tend to be reduced (compare Table 3) but remain significant.

		r	p
Dyspnea Anticipation			
[Δ cue dyspnea vs. cue baseline]			
with Δ intensity	OFC R	-0.42	0.003
with Δ unpleasantness	Midbrain/PAG	-0.33	0.016
	Insula R	0.32	0.018
	Insula L	0.49	<0.001
		0.45	0.001
Dyspnea Perception			
[Δ dyspnea vs. baseline]			
with Δ intensity	OFC R	-0.48	0.001
with Δ unpleasantness	OFC R	-0.43	0.002
	Insula R	0.59	<0.001

Supplementary Table 2. Correlation-coefficients (r) and p-values for correlations of anxiety-scores (as measured by STAI-T) with the beta-values extracted from areas showing significant correlations of brain activation changes with either Δ intensity or Δ unpleasantness. Significant correlations with anxiety are mainly observed for the extracted betas from the cue-period.

	r*	p	$r_{partial}$**	p
Areas showing significant correlations during cue-period				
OFC R	-0.38	0.005	-0.25	0.052
Midbrain/PAG	-0.38	0.005	-0.21	0.09
Insula R	0.45	0.001	0.36	0.008
Insula L	0.27	0.036	0.041	0.4
	0.27	0.036	0.01	0.47
Areas showing significant correlations during dyspnea-period				
OFC R	-0.017	0.46	0.1	0.27
OFC R	0.17	0.13	-0.01	0.47
Insula R	0.14	0.18	-0.18	0.12

*Bivariate correlation-analysis *without* correction for those variables that were included in the partial correlation analysis carried out in SPM (Δ intensity/Δ unpleasantness, P_I and f).
**With Δ intensity/Δ unpleasantness as applicable and breathing-parameters P_I and f as covariates-of-no-interest.
